# Association of Tissue Transglutaminase Antibody Titer with Duodenal Histological Changes in Children with Celiac Disease

**DOI:** 10.1155/2016/6718590

**Published:** 2016-10-27

**Authors:** Hasan Hawamdeh, Basim Al-Zoubi, Yasameen Al Sharqi, Ayman Qasrawi, Yousef Abdelaziz, Maha Barbar

**Affiliations:** ^1^Department of Pediatrics, Hashemite University, P.O. Box 1504514, Zarqa 13115, Jordan; ^2^Department of Pediatrics, Prince Hamzah Hospital, P.O. Box 86, Amman 11118, Jordan; ^3^Department of Pathology, Prince Hamzah Hospital, P.O. Box 86, Amman 11118, Jordan

## Abstract

Celiac disease is usually diagnosed by demonstrating gluten enteropathy in small bowel biopsy. Celiac specific antibodies are used as an initial screening test. The goal of this study is to test the relationship of the anti-tTG titer and severity of histological changes in Jordanian children with celiac disease.* Method*. The medical records of 81 children who had elevated anti-tTG titer and had duodenal biopsies available were retrospectively reviewed.* Result*. Assessing the association of anti-tTG titer with duodenal histopathological changes, 94% of those with high anti-tTG titer (≥180 U/mL) had histological evidence of celiac disease. There was statistically significant positive association between high anti-tTG titer and Marsh grading as 82% of patients with Marsh III had high anti-tTG titer (Chi^2^ 18.5;* P *value 0.00; Odds Ratio 8.5). The fraction of patients with Marsh III who were correctly identified as positive by anti-tTG titer ≥ 180 U/mL was high (sensitivity = 81.6). Moreover, the fraction of patients with anti-tTG titer ≥ 180 U/mL who had Marsh III was also high (positive predictive value = 78.4).* Conclusion*. Anti-tTG titer ≥ 180 U/mL had significant positive association with Marsh III histopathological changes of celiac disease.

## 1. Introduction

Celiac disease (CD) is an autoimmune disorder of the small intestine that occurs in genetically predisposed people of all ages and it is caused by a reaction to gluten found in wheat, barley, and rye [1, 2]. In sensitive individuals, the ingestion of food containing gluten primarily causes inflammation in small bowel mucosa, leading to an increase in small intestinal intraepithelial lymphocytes, crypt hyperplasia, and villous atrophy. These histological changes are graded according to the Marsh Modified Classification. Marsh 0 is the preinfiltrative stage; Marsh I is characterized by normal villous architecture with intraepithelial lymphocyte (IEL) infiltration; Marsh II is identified when the IEL infiltration is accompanied by crypt hyperplasia; and Marsh III is characterized by partial, subtotal, or total villous atrophy in addition to IEL infiltration and crypt hyperplasia [3]. There has been a substantial increase in the prevalence of celiac disease in recent years. Epidemiologic studies have demonstrated that the disease is one of the most common genetic diseases in the world population. It is estimated to affect 1 in 100 people worldwide [4–7]. Women have a higher prevalence rate than men with a ratio of 3 : 1 [8]. The strongest evidence for genetic predisposition is the fact that more than 95% of CD patients express HLA-DQ2 and the remainder express HLA-DQ8 [9, 10].

Symptoms of CD range from the classic features, such as diarrhea, weight loss, and malnutrition, to latent symptoms such as isolated nutrient deficiencies without any gastrointestinal manifestations [11].

Moreover, there are many conditions associated with CD. These are mostly autoimmune diseases, which occur 3–10 times more frequently in CD patients than in the general population. The most common ones of these are diabetes mellitus type 1, Hashimoto thyroiditis, IgA nephropathy, Down syndrome, and Turner syndrome [12–20]. Screening for CD is usually done by serologic testing of such celiac specific antibodies as anti-tissue transglutaminase antibodies (tTG), anti-endomysial antibodies (EMA), and anti-deamidated gliadin derived peptides [21, 22]. The diagnosis is confirmed by duodenal mucosal biopsies, which is considered the golden standard for diagnosis of the disease [5, 23, 24]. Anti-tTG antibody titer is the preferred initial screening test for CD because of its high sensitivity and specificity [7, 21, 25]. A strong correlation between the anti-tTG titer and duodenal histological changes has been demonstrated [26–28]. Moreover, the tests can be used to monitor the response and compliance to a gluten-free diet (GFD) among celiac disease patients [29]. Although a duodenal biopsy remains the gold standard diagnostic test, pediatric endoscopy is not readily available in most hospitals and is an invasive procedure. Accordingly, it is quite useful to test if the elevated anti-tTG titer associates significantly with histological changes of CD, particularly if endoscopy cannot be performed. Few studies have been undertaken to test this association in pediatric CD, especially in the Middle East. The purpose of this retrospective study was to assess the association between anti-tTG titer and histological changes in duodenal biopsies of pediatric patients with CD in Jordan.

## 2. Method

This retrospective study was carried out at Prince Hamzah Teaching Hospital in Amman, the capital of Jordan. The hospital is affiliated with the Faculty of Medicine of the Hashemite University. Prior to the study, we obtained approval from the Science and Ethics Committee. In this study were included a total of 81 samples collected during 42 months from June 2012 to December 2015 from children aged 1 to 13 years (males and females) with elevated anti-tTG titer and duodenal endoscopy evaluation. All of them were on gluten containing diet. IgA anti-tTG antibody was the serological test used in our hospital; it was measured by a human recombinant enzyme-linked immunosorbent assay (ELISA), (Anti-huTransG, Generic Assay, Dahlewitz, Germany). According to the kit used in this study, the upper limit of normal (ULN) is 18 U/mL.

Upper endoscopy under conscious sedation was performed for all patients. At least four biopsy samples were obtained from the duodenal bulb and distal duodenum. Specimens were submerged into a jar containing 10% buffered formalin. The specimens then were stained with hematoxylin and eosin. All biopsies were reviewed and reported independently twice. In the first time, the pathologist reported the biopsies as part of patients' care, and in the second time one pathologist reviewed all biopsies retrospectively and reported changes according to the Marsh classification. Demographic data about gender, age, diagnosis of diabetes mellitus type 1, short stature, thyroid disorders, gastrointestinal manifestations, and family history of CD were collected in addition to initial anti-tTG and Marsh grading of duodenal biopsies. Analysis was done by SPSS 18. Frequencies and percentages were calculated for the different variables; data was analyzed using Student's* t*-test for continuous variables and *χ* ^2^ statistical test and Odds Ratio for categorical variables. The difference was considered significant if *P* value was equal to or less than 0.05. Sensitivity, specificity, positive predictive value (PPV), and negative predictive value (NPV) of the tTG titer ≥ 180 U/mL were calculated as a tool to detect the cases of celiac with Marsh III.

## 3. Results

A total of 81 children were included in this study. The female patients (60.5%) were predominant in sample; mean age at diagnosis was 4.04 years (SD: 3.16 years; range 1–13 years). The mean age of males and females was similar (4.59, SD: 3.09 and 3.67, SD: 3.22, resp.; *P*: 0.2). Demographic and clinical characteristics of the patients are shown in Table 1. Twenty patients (24.7%) had diabetes mellitus (DM) type 1; ten patients (12.3%) had thyroid disease, and thirty-eight patients (46.9%) had at least one gastrointestinal symptom, such as diarrhea, vomiting, or abdominal distension. Short stature was seen in thirty-eight patients (46.9%). Twenty-one patients (25.9%) had a positive family history of celiac disease.

Regarding the histopathological changes, sixty-six patients (81.5%) had histological changes of CD while fifteen patients (18.5%) had normal duodenal biopsies and as such were considered potential CD patients. On the other hand, forty-nine patients of those with histopathological changes (74.2%) had Marsh III, and seventeen patients (25.8%) had Marsh I or II. Fifty-one patients (63%) had an anti-tTG titer ≥ 180 U/mL, and thirty patients (37%) had a titer less than 180 U/mL.

The assessment of association of anti-tTG titer with the histopathological changes of duodenal biopsies is shown in Table 2. Forty of the fifty-one patients (78.4%) with anti-tTG ≥ 180 had Marsh III, eight patients (15.7%) had Marsh I or Marsh II, and only three patients (5.9%) had normal duodenal biopsies (Marsh 0). Regarding the thirty patients with anti-tTG < 180 U/mL, twelve of them (40%) had normal duodenal biopsies (potential CD), while eighteen of them (60%) had histological changes of celiac disease; half of them had Marsh III.

Further analysis to assess the association between the anti-tTG titer and Marsh grading was performed as shown by Table 3. It is obvious that anti-tTG titer and Marsh grading are strongly associated (*χ* ^2^ 18.5; *P* value 0.000) and patients with anti-tTG titer ≥ 180 U/mL are much more likely to have Marsh III compared to those with anti-tTG titer ≤ 180 U/mL (Odds Ratio 8.5).

In order to quantify how good the test is (anti-tTG titer ≥ 180 U/mL) at picking out patients with Marsh III, sensitivity, specificity, positive predictive value (PPV), and negative predictive value (NPV) were calculated as seen in Table 4. The sensitivity was 81.6 which means that 82% of cases with Marsh grading III had anti-tTG titer ≥ 180 U/mL, while 66% of cases with Marsh grading < III had anti-tTG titer < 180 U/mL (specificity = 65.6%).

On the other hand, there about 78% of patients with anti-tTG titer ≥ 180 U/mL were classified as Marsh III (positive predictive value (PPV = 78.4%)), while 70% of cases with tTG titer < 180 U/mL were classified as patients with Marsh grading less than III.

## 4. Discussion

CD specific antibody tests are the initial tools that are used to identify individuals in need of further investigation to diagnose or exclude CD. Systematic review comparing the endomysial (EMA) and tissue transglutaminase (tTG) antibodies concluded that human recombinant tTG IgA antibody is the preferred test for screening asymptomatic people and for excluding celiac disease in symptomatic individuals [30]. The deamidated gliadin derived peptides have shown to be useful monitoring the compliance of the gluten-free diet [31].

Meta-analysis of published studies comparing deamidated gliadin peptide antibody and tissue transglutaminase antibody showed that the tissue transglutaminase antibody test outperforms the deamidated gliadin peptides antibody test and remains the preferred serological test for the diagnosis and/or exclusion of celiac disease [21]. Anti-tissue transglutaminase antibodies have a greater than 95% sensitivity and specificity for celiac disease [32, 33]. Significant association of high anti-tTG titer and histological changes in CD had been reported in many retrospective studies of CD [34–36]. Barker et al. showed that 48 of 49 symptomatic children with an anti-tTG titer ≥ 100 U/mL had at least Marsh II enteropathy [37].

Donaldson et al. showed that all their pediatric patients with anti-tTG titer ≥ 100 U/mL had Marsh III histopathology of CD [26].

The latest European Society of Pediatric Gastroenterology, Hepatology and Nutrition (ESPGHAN) CD guideline in 2012 stated that children and adolescents with signs or symptoms suggestive of CD have high anti-tTG titers with levels >10 times ULN; the likelihood for villous atrophy (Marsh III) is high, so duodenal biopsy can be omitted if this antibody positivity is verified by positive EMA and/or HLA DQ2/DQ8 heterodimer [33]. Following the 2012 ESPGHAN guideline, studies were done to assess validity of the guideline. Gidrewicz et al. showed that 98% of symptomatic pediatric patients with anti-tTG ≥ 10 times ULN and positive EMA had biopsies consistent with celiac disease [38]. Our study similarly showed that anti-tTG equal to or more than 10 times ULN was significantly correlated with histopathological changes of CD as 94% of our patients with anti-tTG ≥ 10 times ULN had histological evidence of CD. Also, a significant correlation was reported between high anti-tTG titer and higher Marsh grade [39]. Similarly, our data showed forty of the fifty-one patients with anti-tTG ≥ 10 times ULN had Marsh III (78.4%; *χ* ^2^ = 18.5; *P* value = 0.000; and Odds Ratio = 8.5) (Table 3).

Trovato et al. evaluated the ESPGHAN biopsy-sparing guideline in symptomatic and asymptomatic celiac disease and reported no difference histologically between high titer symptomatic and high titer asymptomatic groups as Marsh III was demonstrated in 91% and 92% of these patients, respectively [40].

Stratifying our patients into two groups, thirty-eight patients (47%) had gastrointestinal manifestations, and forty-three patients (53%) did not. Assessing the histological differences between the two groups, there was no statistically significant difference as twenty-four patients (63%) with gastrointestinal manifestations had Marsh III and twenty-five patients (58%) with no gastrointestinal manifestations had Marsh III (*P* value 0.41). Moreover, the difference in the mean anti-tTG titer was not statically significant between the two groups (*t*-test 0.28, *P* value 0.78).

Although significant association between high titer and severity of duodenal histology was demonstrated by our study, there were nine patients (18%) that had Marsh III histology but their tTG level though elevated <10 times ULN. The reason might be the probability of variable gluten intake or the variable ability of patients to mount antibody response. This observation highlights importance of assessing gluten intake and total IgA level when interpreting the tTG titer.

Our observation is supported by Lewis and Scott who reported 5–16% of biopsy confirmed celiac disease had negative tTG titer [21]. Three (20%) of our patients with Marsh 0 had tTG titer > 10 times ULN, and those met the definition of potential celiac; two of those patients had diabetes, and many studies reported that false positive results may occur in patients with other autoimmune diseases. Other possible explanation is the fact that histological abnormalities in celiac disease can be patchy [24, 41–44].

This is similar to the report of Freeman which showed that 20% of patients with anti-tTG > 100 U/mL had a normal biopsy [45]. Those patients are candidates for repeating duodenal endoscopy and/or HLA testing if celiac disease as a diagnosis is highly suspected.

It was not possible to enhance the diagnostic value of elevated anti-tTG results in our patients with anti-EMA or HLA analysis as those tests are not available at our laboratory.

Because of the small size of our study population, it was not possible to further analyze other variables in relation to anti-tTG titer and histological changes, such as autoimmune disease status, positive family history of CD, or short stature.

Our study has several limitations, including the small number of patients and the unavailability of anti-EMA and HLA tests, but it clearly supports the significant association between high tTG titer and the severity of duodenal histological damage.

## 5. Conclusion

Our study showed that there was a significant association between anti-tTG titers and the degree of duodenal damage. An anti-tTG titer more than 10 times the ULN (anti-tTG titer ≥ 180 U/mL) was significantly associated with Marsh III enteropathy. Although demonstrating histological changes of gluten, enteropathy is still the standard diagnostic test of CD; this strong association of high anti-tTG titer and severity of histological changes might supply sufficient evidence for CD diagnosis when supported by positive EMA or HLA test when endoscopy is not feasible.

## Figures and Tables

**Table 1:** Demographic and clinical characteristics of patients.

Variable	Frequency	Percentage
Sex		
Female	49	60.5
Male	32	39.5

Diabetes		
Yes	20	24.7
No	61	75.3

Hypothyroidism		
Yes	10	12.3
No	71	87.7

Gastrointestinal symptoms		
Yes	38	46.9
No	43	53.1

Short stature		
Yes	38	46.9
No	43	53.1

Family history of celiac		
Yes	21	25.9
No	60	74.1

Histopathology changes		
Yes	66	81.5
No (silent)	15	18.5

Marsh grading of histopathology changes		
Marsh 0	15	18.5
Marsh I	3	3.7
Marsh II	14	17.3
Marsh III	49	60.5

tTG titer		
<180	30	37
≥180	51	63

**Table 2:** Marsh classification and anti-tTG titer cross tabulation.

Marsh classification	Anti-tTG titer		Total
	<180 U/mL No. (%)	≥180 No. (%)	
0	12 (40)	3 (5.9)	15
I and II	9 (30)	8 (15.7)	17
III	9 (30)	40 (78.4)	49

Total	30	51	81

**Table 3:** Association between Marsh grading and tTG titer.

Variable	Anti-tTG titer		Total	*Pearson χ* ^2^	*P* value	Odds Ratio
	<180 No.	≥180 No.				
Marsh grading				18.5	0.000	8.5
<III	21	11	32			
III	9	40	49			

Total	30	51	81			

**Table 4:** Assessment of sensitivity, specificity, positive predictive value, and negative predictive value of the anti-tTG titer ≥ 180 U/mL.

Test	Marsh III	<Marsh III	Total
Anti-tTG ≥ 180 U/mL (positive)	40	11	51
Anti-tTG < 180 U/mL (negative)	9	21	30

Sensitivity 81.6%, specificity 65.6%, PPV 78.4%, NPV 70%.
