# Erratum to “Association of Tissue Transglutaminase Antibody Titer with Duodenal Histological Changes in Children with Celiac Disease”

**DOI:** 10.1155/2019/2607194

**Published:** 2019-04-14

**Authors:** Hasan Hawamdeh, Basim Al-Zoubi, Yasameen Al Sharqi, Ayman Qasrawi, Yousef Abdel-Aziz, Maha Barbar

**Affiliations:** ^1^Department of Pediatrics, Hashemite University, P.O. Box 1504514, Zarqa 13115, Jordan; ^2^Department of Pediatrics, Prince Hamzah Hospital, P.O. Box 86, Amman 11118, Jordan; ^3^Department of Pathology, Prince Hamzah Hospital, P.O. Box 86, Amman 11118, Jordan

In the article titled “Association of Tissue Transglutaminase Antibody Titer with Duodenal Histological Changes in Children with Celiac Disease” [1], the name of the fifth author was given incorrectly as Yousef Abdelaziz. The author's name should have been written as Yousef Abdel-Aziz. The revised authors' list is shown above.
